# Maximising BOLD sensitivity through automated EPI protocol optimisation

**DOI:** 10.1016/j.neuroimage.2018.12.052

**Published:** 2019-04-01

**Authors:** Steffen Volz, Martina F. Callaghan, Oliver Josephs, Nikolaus Weiskopf

**Affiliations:** a Department of Neurophysics, Max Planck Institute for Human Cognitive and Brain Sciences, Leipzig, Germany; b Wellcome Centre for Human Neuroimaging, UCL Institute of Neurology, University College London, London, UK

## Abstract

Gradient echo echo-planar imaging (GE EPI) is used for most fMRI studies but can suffer substantially from image distortions and BOLD sensitivity (BS) loss due to susceptibility-induced magnetic field inhomogeneities. While there are various post-processing methods for correcting image distortions, signal dropouts cannot be recovered and therefore need to be addressed at the data acquisition stage. Common approaches for reducing susceptibility-related BS loss in selected brain areas are: z-shimming, inverting the phase encoding (PE) gradient polarity, optimizing the slice tilt and increasing spatial resolution. The optimization of these parameters can be based on atlases derived from multiple echo-planar imaging (EPI) acquisitions. However, this requires resource and time, which imposes a practical limitation on the range over which parameters can be optimised meaning that the chosen settings may still be sub-optimal. To address this issue, we have developed an automated method that can be used to optimize across a large parameter space. It is based on numerical signal simulations of the BS loss predicted by physical models informed by a large database of magnetic field (B_0_) maps acquired on a broad cohort of participants. The advantage of our simulation-based approach compared to previous methods is that it saves time and expensive measurements and allows for optimizing EPI protocols by incorporating a broad range of factors, including different resolutions, echo times or slice orientations. To verify the numerical optimisation, results are compared to those from an earlier study and to experimental BS measurements carried out in six healthy volunteers.

## Highlights

• Significant BOLD sensitivity increase by optimization of z-shim, gradient polarity and slice tilt.
• Method based on numerical signal simulations informed by large database of magnetic field maps.
• Saves time and expensive measurements.
• Automated and flexible optimization of multiple EPI parameter settings.

## 1 Introduction

Gradient echo echo-planar imaging (GE EPI) (Mansfield, 1977) is used for most functional magnetic resonance imaging (fMRI) studies due to its high acquisition speed and its sensitivity to the blood oxygenation level-dependent (BOLD) effect (Ogawa et al., 1990).

However, EPI quality can suffer substantially from image distortions and BOLD sensitivity loss (BS) caused mainly by magnetic field inhomogeneities. These inhomogeneities originate from differences in the magnetic susceptibility between tissue and air and are especially prominent in the orbitofrontal cortex (OFC), the medial temporal and the inferior temporal lobes (Ojemann et al., 1997; Devlin et al., 2000; Lipschutz et al., 2001).

The correction of image distortions is mostly performed during image post-processing and there exist various methods (Andersson et al., 2001; Bowtell et al., 1994; Hutton et al., 2002; Jezzard and Balaban, 1995; Sutton et al., 2004; Weiskopf et al., 2005; Zaitsev et al., 2004; Mohammadi et al., 2010). However, the loss of BS still remains and needs to be addressed at the data acquisition stage.

For reducing susceptibility-related signal losses a variety of techniques have been introduced. For example, the differences in susceptibility can be reduced directly by placing diamagnetic materials with susceptibilities similar to tissue around the participant (Fritzsche et al., 1995) or using oral shim coils (Hsu and Glover, 2005; Wilson and Jezzard, 2003). However, such shimming is limited to a relatively small area and situations with strong susceptibility gradients. Furthermore, additional hardware and increased manual effort are needed, potentially also causing additional inconvenience to the patient or volunteer. Alternatively, susceptibility-induced signal losses can be reduced by the use of 3D tailored radiofrequency (RF) pulses (Stenger et al., 2000; Yip et al., 2006; Zheng et al., 2013) or spectral-spatial pulses (Anderson et al., 2014; Yip et al., 2009) without the need for additional hardware. However the design of these pulses is computationally expensive and leads to prolonged RF pulse durations, hence echo time TE and repetition time TR, and often reduce signal-to-noise-ratio (SNR) in well-shimmed areas (Cho and Ro, 1992).

Various strategies have been developed for compensating susceptibility-induced gradients in selected brain areas. The z-shimming approach (Frahm et al., 1988; Ordidge et al., 1994; Posse et al., 1999; Deichmann et al., 2003; Rick et al., 2010) compensates gradients in the slice direction. This has been extended to in-plane gradients in the phase-encoding (PE) direction (Deichmann et al., 2003; De Panfilis and Schwarzbauer, 2005) and to the readout (RO) direction (Weiskopf et al., 2007). One drawback of adding compensation gradients to the sequence however is the reduction of spatial and temporal resolution. Other approaches that avoid prolonging the acquisition significantly are optimising the slice tilt (De Panfilis and Schwarzbauer, 2005; Weiskopf et al., 2006) and TE (Domsch et al., 2013; Stöcker et al., 2006). Signal dropouts can also be improved by multi-echo summation (Posse et al., 1999), increasing the spatial resolution (Robinson et al., 2004; Weiskopf et al., 2007), using thin slices (Frahm et al., 1993; Merboldt et al., 2000) or combining high resolution with high acquisition speed by parallel imaging (Heidemann et al., 2012; Domsch et al., 2015). The poor temporal resolution of high resolution acquisition techniques can additionally be improved by multiband EPI sequences (Nunes et al., 2006; Setsompop et al., 2012) shown to improve signal dropouts in Kim et al. (2016).

The optimisation of all these parameters can be based on atlases derived empirically from multiple EPI acquisitions (Weiskopf et al., 2006). This however is resource and time consuming. Thus, atlases reporting voxel-wise optimal sequence parameters cover a limited set of parameters, e.g. z-shim, the gradient polarity and the slice tilt, over a relatively coarse range and with a particular acquisition protocol, e.g. oblique transverse EPI acquisition at 3 mm resolution only (Weiskopf et al., 2006).

In the present work we develop and employ a flexible and automated BS optimization method that is based on the prediction of BS loss using the physical models accounting for through-plane dephasing and local echo time/k-space shifts and signal loss due to susceptibility-induced in-plane gradients in the PE (Deichmann et al., 2003; De Panfilis and Schwarzbauer, 2005) and RO direction (Weiskopf et al., 2007). A database of magnetic (B_0_) field maps acquired over a large population of 138 volunteers serves as input for the calculations. Unlike the previous experimental approaches, the presented approach allows arbitrary 2D-EPI acquisition protocols, including varying resolution, echo time or slice orientation, to be optimised making it more easily and widely applicable.

## 2 Methods

### 2.1 BS calculation

The BS is defined as the local signal intensity change due to a local change of the effective transverse relaxation time T_2_^∗^ being altered during neuronal activation. Given the local echo time TE and the signal intensity I, it can be calculated according to Lipschutz et al. (2001):

(1) BS=TE⋅I

In the following I_0_ʹ denotes the initial signal intensity at TE = 0 and TE_0_ and I_0_ denote the respective echo time and initial signal intensity if there are no susceptibility gradients.

The signal intensity I of a gradient echo EPI sequence is given by:

(2) $$I=I_{0}^{'}\cdot \text{exp}\left(-\frac{TE}{T_{2}^{*}}\right)$$

A susceptibility gradient in the slice direction G_S_^susc^ causes through-plane spin dephasing and thus a signal loss. It can be compensated with a z-shim gradient G_S_^shim^ (Frahm et al., 1988; Ordidge et al., 1994; Deichmann et al., 2003; Rick et al., 2010) applied with opposite polarity in the slice direction for a time period τ. For a Gaussian-shaped excitation profile and a slice thickness of Δz, the corresponding image intensity is given according to Deichmann et al. (2002) by:

(3) $$I=I_{0}\cdot \text{exp}\left(-{\text{Ψ}}^{2}\right),with\cdot \text{Ψ}=\text{γ}\cdot \frac{\text{Δ}z}{4\sqrt{\ln\left(2\right)}}\cdot \left(G_{S}^{susc}\cdot TE+G_{S}^{shim}\cdot \tau \right)$$

γ is the gyromagnetic ratio. Susceptibility gradients along in-plane directions sum up with the imaging gradients causing distortion of the data in k-space. A susceptibility gradient in the PE direction G_P_^susc^ causes a change of the local TE and the modified initial signal intensity Iʹ (Deichmann et al., 2002) according to:

(4) $$TE=\frac{TE_{0}}{Q},I^{'}=\frac{I_{0}^{'}}{Q},with,Q_{\pm }=1\mp \frac{\text{γ}\cdot \text{Δt}}{2\pi }\cdot FoV_{P}\cdot G_{P}^{susc}$$

FoV_P_ is the field of view in phase encoding direction and Δt the inter-echo spacing during the EPI readout. The sign in the subscript of Q refers to the PE gradient polarity of the EPI readout and, as referred to later, is defined by the polarity of the PE prewinder moment used in the EPI sequence. A positive PE gradient polarity corresponds to a positive PE prewinder moment (and thus negative phase blip gradients) thus pointing from the posterior to the anterior part of the brain in the example of the transverse acquisition.

Equations (1)–(4) combine to:

(5) $$BS=\frac{BS_{0}}{Q^{2}}\cdot \text{exp}\left(-\frac{TE-TE_{0}}{T_{2}^{*}}\right)\cdot \text{exp}\left(-{\text{Ψ}}^{2}\right)$$

Both in-plane susceptibility gradients in PE direction and in RO direction can shift the centre of k-space outside the acquisition window and therefore cause a complete signal dropout. For susceptibility gradients in the PE direction, in the case of symmetric k-space sampling with sampling duration TA of the EPI readout, the following condition has to be fulfilled (Deichmann et al., 2003) to prevent signal dropout:

(6) $$TE_{0}-\frac{TA}{2}\leq TE\leq TE_{0}+\frac{TA}{2}$$

For a susceptibility gradient in the RO direction G_R_^susc^ in order for the k-space shift ΔK_susc_ not to exceed the acquisition window in the RO direction the following condition has to be fulfilled if Δ x is the RO resolution (Weiskopf et al., 2007):

(7) $$\left|\text{Δ}K_{susc}\right|=\left|\text{γ}\cdot TE\cdot G_{R}^{susc}\right|\leq \frac{\pi }{\text{Δx}}$$

If one of the conditions (6) or (7) is not fulfilled, this results in a complete signal loss and BS = 0.

### 2.2 Acquisition of a large magnetic field map database

The BS simulations rely on accurate estimates of the typical static magnetic field distribution in the brain. B_0_ field maps from 138 healthy volunteers (49 men, age range 19–75 years, age mean ± standard deviation 46.6 ± 21 years), who were scanned as part of the neuroscience research program at our imaging centre (Wellcome Centre for Human Neuroimaging; WCHN) with Ethics approval, served as an estimate for a population of healthy volunteers. The field maps were acquired using a double echo FLASH sequence with the following parameters: 64 transverse slices, slice thickness = 2 mm, gap between slices = 1 mm, TR = 1020 ms, α = 90°, short TE = 10 ms, long TE = 12.46 ms, BW = 260 Hz/pixel, PE direction right–left, FOV = 192 × 192 mm^2^, matrix size 64 × 64, flow compensation. In addition to the field maps, anatomical data (3D FLASH) were recorded for each volunteer as part of a whole brain quantitative multi-parameter mapping (MPM) protocol (Callaghan et al., 2014). All data were acquired on a 3 Tesla whole body MR scanner (Magnetom TIM TRIO, Siemens Medical Solutions, Erlangen, Germany) using the standard 32 channel head coil for RF receive and the RF body coil for transmission. Informed written consent was obtained from each volunteer prior to scanning.

### 2.3 Data pre-processing and BS calculation

All data were analysed and processed with Matlab (MathWorks, Natick, MA) and SPM8 (http://www.fil.ion.ucl.ac.uk/spm) and custom-written Matlab programs. Field maps were estimated from the GE data using the Field Map toolbox (Hutton et al., 2002). Field gradients were derived from the field maps by numerical differentiation and normalised to MNI space using the individual anatomical data using Dartel (Ashburner, 2007). No modulation and smoothing were applied to preserve the values. After normalization they were averaged. Based on this, BS maps were calculated according to Equation (5). Additionally, BS maps were calculated for each subject separately, providing an estimate of the variability of BS changes and optimal parameters across the population.

### 2.4 Optimization of BS

The optimization was carried out by stepping through all parameters within the ranges as described further down to find the EPI parameters maximizing the BS. This optimisation was carried out first voxel-wise across the whole brain. Then, separate optimisations were done for the following regions of interest (ROIs) by maximizing the mean BS across the respective ROIs: (1) medial orbitofrontal cortex (mOFC) and rostral–ventral anterior cingulate cortex (rACC), (2) inferior temporal lobes, (3) temporal poles, (4) amygdala and (5) hippocampus and para-hippocampus. The ROIs were based on the automated anatomical labeling toolbox (Tzourio-Mazoyer et al., 2002) and were constructed to compare with previous work as described in detail in Weiskopf et al. (2006). Optimized EPI parameter sets were only accepted if the BS loss in areas not affected by susceptibility related gradients, i.e. for a susceptibility gradient with zero value, did not exceed 15% (Weiskopf et al., 2006) compared to the standard EPI. Otherwise, the EPI parameter set with the next highest BS not exceeding the 15% BS loss was chosen. In other words, the 15% cut off served as a hard boundary condition for the optimization.

While the simulations can be carried out for arbitrary 2D-EPI protocols, for conciseness we determined the optimal PE polarity, slice angulation and in-plane rotation and through plane z-shim gradients for the following reference EPI protocols with settings typical of studies carried out at the WCHN. Since particular slice orientations impose different restrictions on the spatial coverage that can be achieved, the three primary planes (transverse, sagittal, coronal) were investigated separately. The reference protocols are:

(I) **Standard resolution protocol:** TE = 30 ms, echo spacing = 0.5 ms, in-plane resolution = 3 × 3mm^2^, slice thickness = 3 mm, matrix size = 64 × 64, no acceleration by parallel imaging or partial Fourier each for transverse, sagittal and coronal slices respectively with PE gradient directions pointing from anterior to posterior for transverse and sagittal slices and feet to head for coronal slices.
(II) **High-resolution protocol:** TE = 30 ms, echo spacing = 0.78 ms, in-plane resolution = 2 × 2mm^2^, slice thickness = 2 mm, matrix size = 96 × 96, no acceleration by parallel imaging or partial Fourier again for transverse, sagittal and coronal slices respectively with PE gradient directions pointing from anterior to posterior for transverse and sagittal slices and feet to head for coronal slices.
(III) **Accelerated high-resolution protocol:** This protocol was identical to (II) but a speedup using GRAPPA with factor 2 in the PE direction was simulated.

The ranges used for each of the parameters to be optimised were: positive or negative polarity of the PE gradient, slice tilt ranging from −45° to 45° in steps of 5°, and z-shim gradient pointing in slice direction with a moment ranging from −5 to 5 mT/m*ms in steps of 0.5 mT/m*ms.

**Fig. 1:**
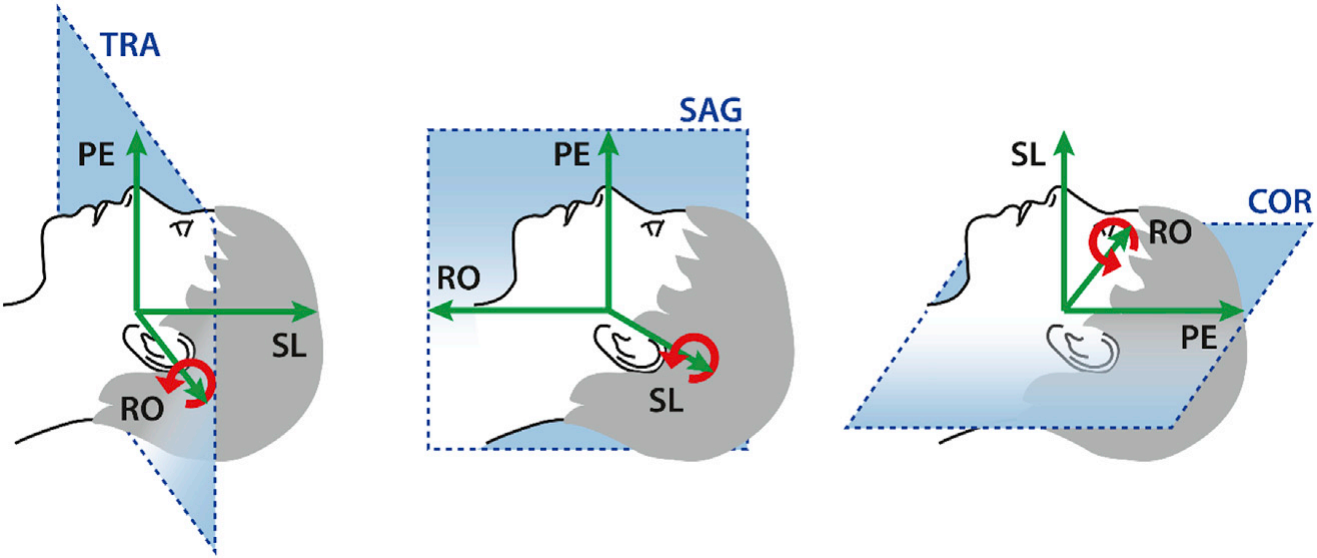
Definition of coordinate axis for phase encoding (PE), readout (RO) and slice direction (SL) for the main slice orientations transverse (left), sagittal (middle) and coronal (right) as used in the experiments. For illustration of slice orientations, slices are overimposed over a sagittal view of the human head. The directions for slice angulations and in-plane rotation are denoted with the red arrows respectively.

Fig. 1 illustrates the definitions of the coordinate systems for the different main slice orientations and the corresponding directions for slice angulations and in-plane rotations, respectively. For example, in case of the transverse acquisition a slice angulation means a rotation around the RO axis which is pointing from the right to the left hemisphere of the brain with a positive rotation angle denoting an angulation of the anterior part of the slices from head to feet.

### 2.5 Validation by comparison to published approaches

The simulation results were compared to results obtained from measured data reported in Weiskopf et al. (2006). A BS optimization was performed with the same fixed sequence parameters and parameter range for the simulation as used in the measurements at 3T. The following fixed EPI imaging parameters were used: TE = 30 ms, slice thickness = 2 mm, in-plane resolution = 3 × 3mm^2^, echo spacing = 0.33 ms, matrix size = 64 × 64, transverse slices and the PE gradient directions pointing from anterior to posterior. The following parameters were optimized for the ROIs listed above and varied in the same range as in the publication: slice tilt ranging from −45° to 45° in steps of 15°, z-shim gradient pointing in slice direction with a moment ranging from −4 to 4 mT/m*ms in steps of 1 mT/m*ms and the polarity of the PE gradient pointing either in positive or negative direction.

### 2.6 Validation by comparison to in vivo data

The simulation output was compared to experimental data from in vivo measurements. Six volunteers (four male, age range 27–38 years, age mean ± sd = 33 ± 4 years) were scanned with various slice tilts, z-shims, PE gradient polarity and slice orientation on a 3T Tim Trio MRI scanner. The parameters of the EPI protocol were fixed to match reference protocol (1) above. The other parameters were: 48 slices, TR = 3360 ms and 13% phase oversampling (acquired k-space lines = 64 × 74). A B_0_ field map (as described above for the database creation) was acquired for distortion correction of the EPI images and for simulating the BS and a short anatomical scan (3D MP-RAGE sequence with 1 mm isotropic resolution, field-of-view = 176 × 224 × 256 mm^3^ and TI/α/TE/TR = 900 ms/9°/2.26 ms/1900 ms) were also acquired. Informed written consent was obtained from each volunteer before participating and the study was approved by the local Ethics committee.

Data were acquired using a circularly polarized (CP) head coil for RF receive and transmission in order to facilitate reconstruction of robust magnitude and phase data avoiding multi-channel image reconstruction.

The measurement was repeated 36 times with different combinations of main slice orientations (transverse and sagittal), slice tilts/in plane rotations (−30, 0 and 30°), z-shim (through plane) gradient moments (−3, 0 and 3 mT/m*ms) and PE gradients with a positive and negative polarity. For each measurement, 6 vol were acquired, and both magnitude and phase images were exported for later evaluation.

For each EPI acquisition and each volunteer, experimental BS maps were calculated from the complex image data using the method described in appendix C of Deichmann et al. (2002) for determining the local TE via the phase difference between two adjacent points in the PE direction. The BS was then calculated as the product of this local TE and the magnitude of the image intensity I: BS = TE*I. These BS maps were undistorted using the Field Map toolbox (Hutton et al., 2002). BS gain maps, in percent units, were calculated voxel-wise according to BS_gain,exp_ = 200*(BS-BS_ref_)/(BS + BS_ref_) using the default protocol, i.e. with no slice tilt, zero z-shim gradients and a positive polarity of the PE gradient as reference BS_ref_.

For comparison, the BS was simulated for each EPI protocol and each volunteer, using the individual's specific field map. Similar to the calculation above expected BS gain maps, in percent units, were calculated according to BS_gain,sim_ = 200*(BS-BS_ref_)/(BS + BS_ref_) from the simulation using the default protocol, i.e. with no slice tilt, zero z-shim gradient and a positive polarity of the PE gradient as reference BS_ref_.

In order to compare the simulated and experimental BS gain only within the brain, a brain mask was obtained by segmenting the MP-RAGE scan, summing the WM, GM and CSF tissue probabilities and thresholding at 0.95. Each of the simulated BS maps calculated for each individual were co-registered to the experimental EPI data acquired with the default protocol and then the difference between the simulated and experimental BS gain BS_diff_ = BS_gain,sim_-BS_gain,exp_ was calculated. Mean and standard deviation of the BS gain difference were evaluated within the brain mask.

## 3 Results

### 3.1 Field gradients in 138 volunteers

**Fig. 2:**
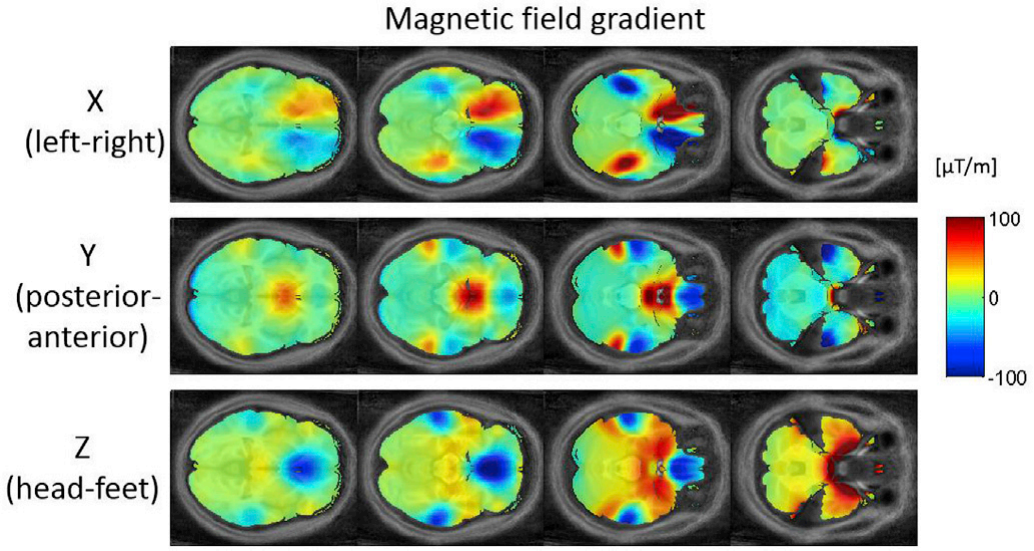
Maps of susceptibility-induced B_0_ field gradients obtained from the field maps for all three directions.

Fig. 2 shows maps of the magnetic field gradients along the X-direction (left-right, top row), Y-direction (posterior-anterior, middle row) and Z-direction (head-feet, bottom row). Maximum gradients, of up to ±100 μT/m were found in the temporal and orbitofrontal areas. While the contribution of gradients in Y— and Z-direction were found to be left-right symmetric, an antisymmetric (same magnitude, but opposite sign) contribution of gradients in X-direction was seen. Field gradients in Y-direction with opposing signs close to each other were observed in the orbitofrontal and temporal areas.

### 3.2 Results for optimization of BS

**Fig. 3:**
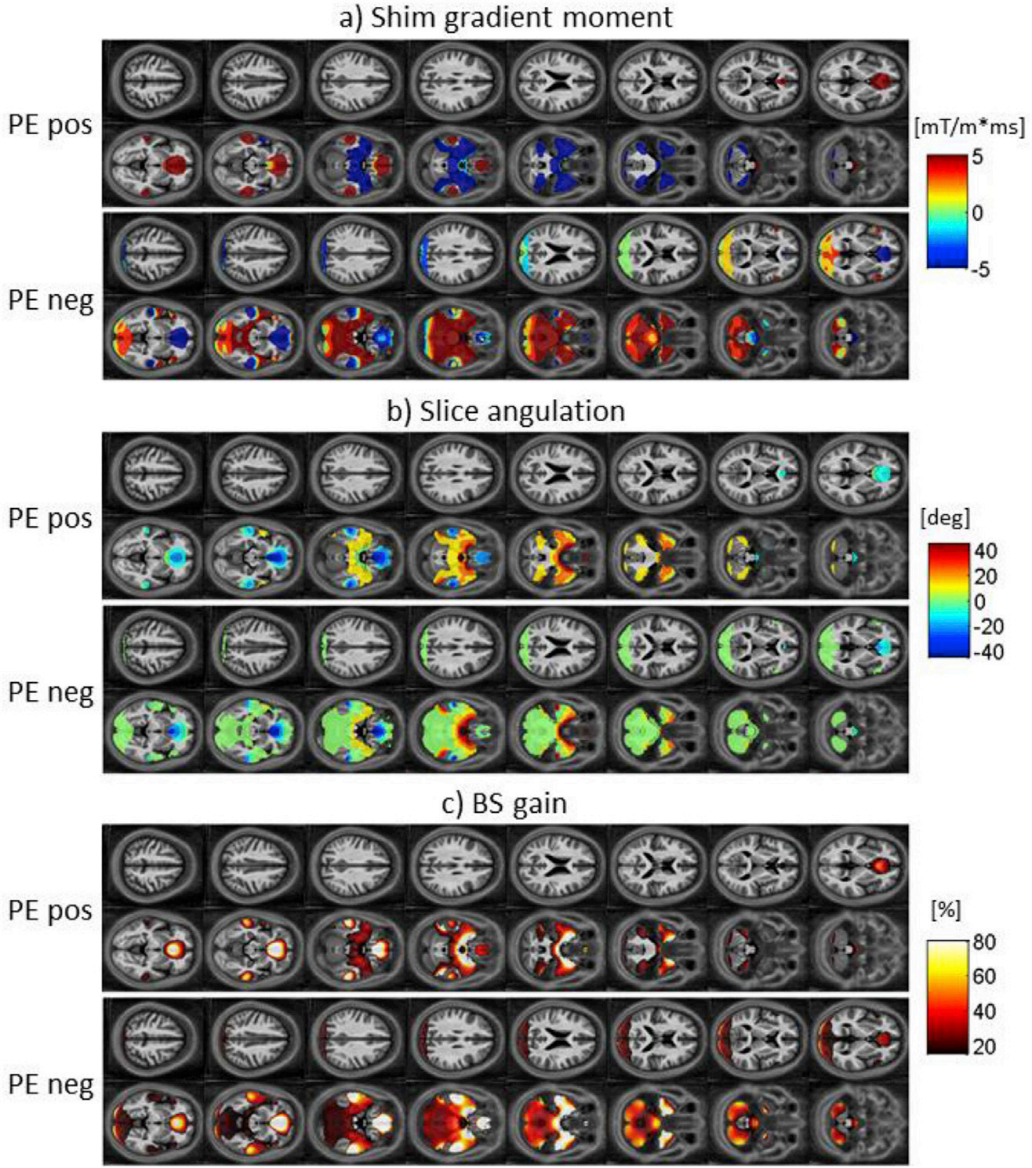
Maps of the voxel-wise optimized parameters for 16 slices in case of oblique transverse acquisitions with an in-plane resolution of 3 × 3mm^2^, a matrix size of 64 × 64 and a slice thickness of 3 mm. Optimal shim gradient moment (a), optimal slice angulation (b) and BS gain achieved with the optimal parameter set compared to standard EPI with no shim gradient and slice angulation (c). In each case the optimized parameters are shown for a positive PE gradient (top row) and for a negative PE gradient (bottom row). A mask has been applied to show only optimized parameters with a BS gain of at least 20%.

**Fig. 4:**
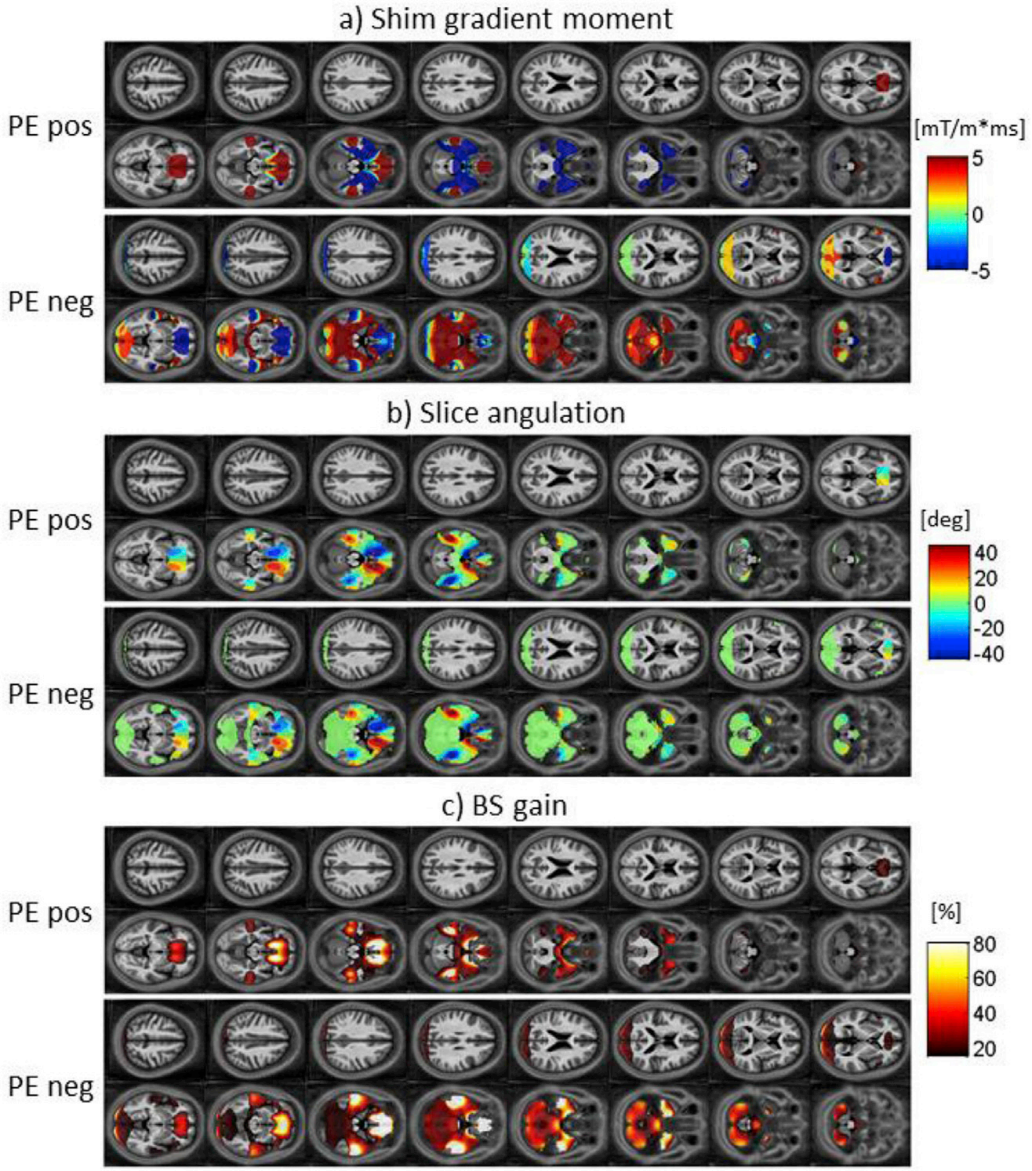
Maps of the voxel-wise optimized parameters for 16 slices in case of sagittal acquisitions. Optimal shim gradient moment (a), optimal in-plane rotation (b) and BS gain achieved with the optimal parameter set compared to standard EPI with no shim gradient and slice rotation (c). In each case the optimized parameters are shown for a positive PE gradient (top row) and for a negative PE gradient (bottom row).

**Fig. 5:**
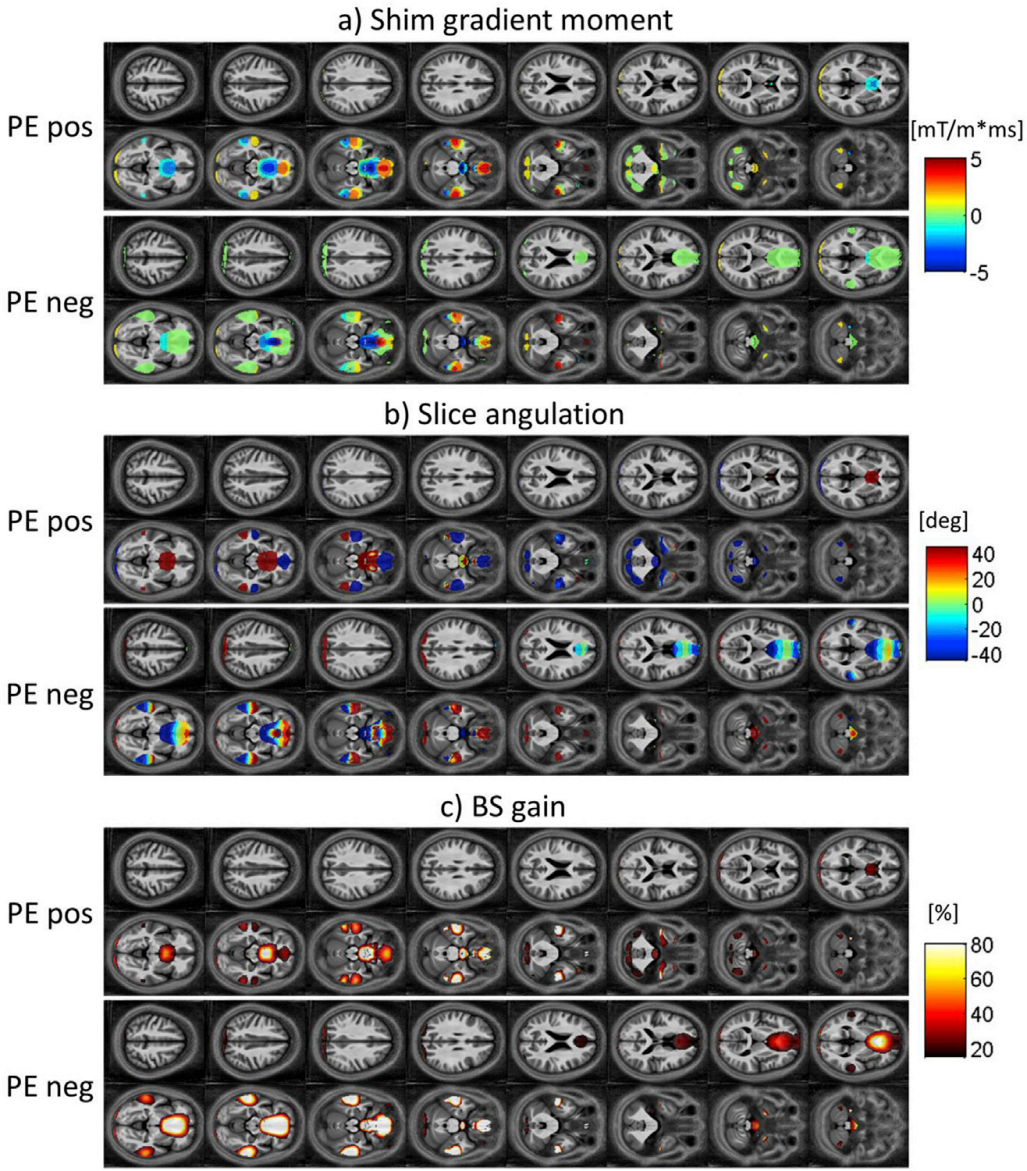
Maps of the voxel-wise optimized parameters for 16 slices in case of coronal acquisitions. Optimal shim gradient moment (a), optimal slice angulation (b) and BS gain achieved with the optimal parameter set compared to standard EPI with no shim gradient and rotation (c). In each case the optimized parameters are shown for a positive PE gradient (top row) and for a negative PE gradient (bottom row).

Fig. 3 shows maps of the voxel-wise optimized parameters for the case of the *standard resolution protocol I* with a transverse slice orientation. Shown are, from top to bottom, maps of the optimal z-shim gradient moment (a), the optimal slice angulation (b) and the maximum BS gain with the optimal z-shim gradient and slice angulation compared to the standard EPI protocol with no shim gradient or slice angulation (c). The results are shown for both a positive PE gradient (top row) and a negative PE gradient (bottom row) respectively. Similarly, Fig. 4, Fig. 5 show the optimized maps for the *standard resolution protocol I* acquisitions with sagittal and coronal orientation respectively.

For the optimal z-shim gradient, a negative gradient moment was found to yield the highest BS in the orbitofrontal cortex and a positive gradient moment in the temporal lobes in case of the transverse acquisition (Fig. 3a) as previously reported for the measurements with a negative PE gradient. For the sagittal acquisition, a left-right antisymmetric distribution of z-shim gradient moments was found in the orbitofrontal cortex and the temporal lobe (Fig. 4a). Combined with the findings in the BS gain the symmetry and antisymmetry can be explained by the susceptibility-induced field inhomogeneities that mainly point symmetrically along the head-feet direction and antisymmetrically in the left-right direction near these areas (Fig. 2). For the coronal acquisition, areas with positive and negative z-shim gradients were found close to each other in the orbitofrontal cortex and the temporal lobe (Fig. 5a).

For the optimal slice angulations/in-plane rotations the optimization results in positive values for transverse/sagittal acquisition in the orbitofrontal cortex and negative values in the temporal lobes. Like for the optimized z-shim gradient moments, areas with positive and negative slice angulations could be found close to each other in the orbitofrontal cortex and temporal lobes in the coronal acquisition case. The similar values for slice angulations and rotations for transverse and sagittal acquisitions are driven by the same PE direction for both acquisitions. Similar to the results for the z-shim gradients, areas with positive and negative values for the slice angulations were found close to each other in the orbitofrontal cortex and the temporal lobe in case of the coronal acquisition (Fig. 5b) according to the field gradient distribution with opposite signs close to each other in the posterior-anterior direction (Fig. 2).

The results of the optimization based on different ROIs (mOFC and rACC, inferior temporal lobes, temporal poles, amygdala and Hippocampus and Parahippocampus), listed in Table 1, reflect the findings presented in Fig. 3, Fig. 4, Fig. 5: positive shim gradients in the temporal lobes but negative in the orbitofrontal cortex in the case of the transverse acquisition in the PE neg scheme. Z-shim values near zero were found for the same regions for the sagittal and coronal acquisitions reflecting the symmetry and antisymmetry of the field inhomogeneities and opposing values for the shim gradients close to each other within these areas.

**Table 1:** Optimal parameters for the three principal orientations: transverse (TRA), sagittal (SAG) and coronal (COR). The basic EPI parameters were: In-plane resolution of 3 × 3 mm^2^, a matrix size of 64 × 64 and a slice thickness of 3 mm. As a measure of generalizability, the standard deviation for the BS-gain across subjects is listed.

Region of interest	Z-shim [mT/m*s]			Tilt [deg]			PE polarity			BS-gain [%]		
Acquisition Protocol	TRA	SAG	COR	TRA	SAG	COR	TRA	SAG	COR	TRA	SAG	COR
mOFC + rACC	−0.5	0.0	0.0	−45	−45	25	Neg	Neg	Neg	37 ± 17	28 ± 11	38 ± 16
Inferior temporal lobes	0.0	0.0	0.0	45	45	−40	Neg	Neg	Pos	12 ± 6	15 ± 6	4 ± 4
Temporal poles	1.0	0.0	0.0	45	45	−5	Neg	Neg	Pos	44 ± 17	27 ± 11	0 ± 3
Amygdala	1.5	0.0	0.0	−45	−45	5	Pos	Pos	Pos	44 ± 23	22 ± 11	0 ± 4
Hippocampus + Parahippocampus	0.5	0.0	0.0	45	45	−15	Neg	Neg	Pos	30 ± 9	22 ± 6	1 ± 2

**Fig. 6:**
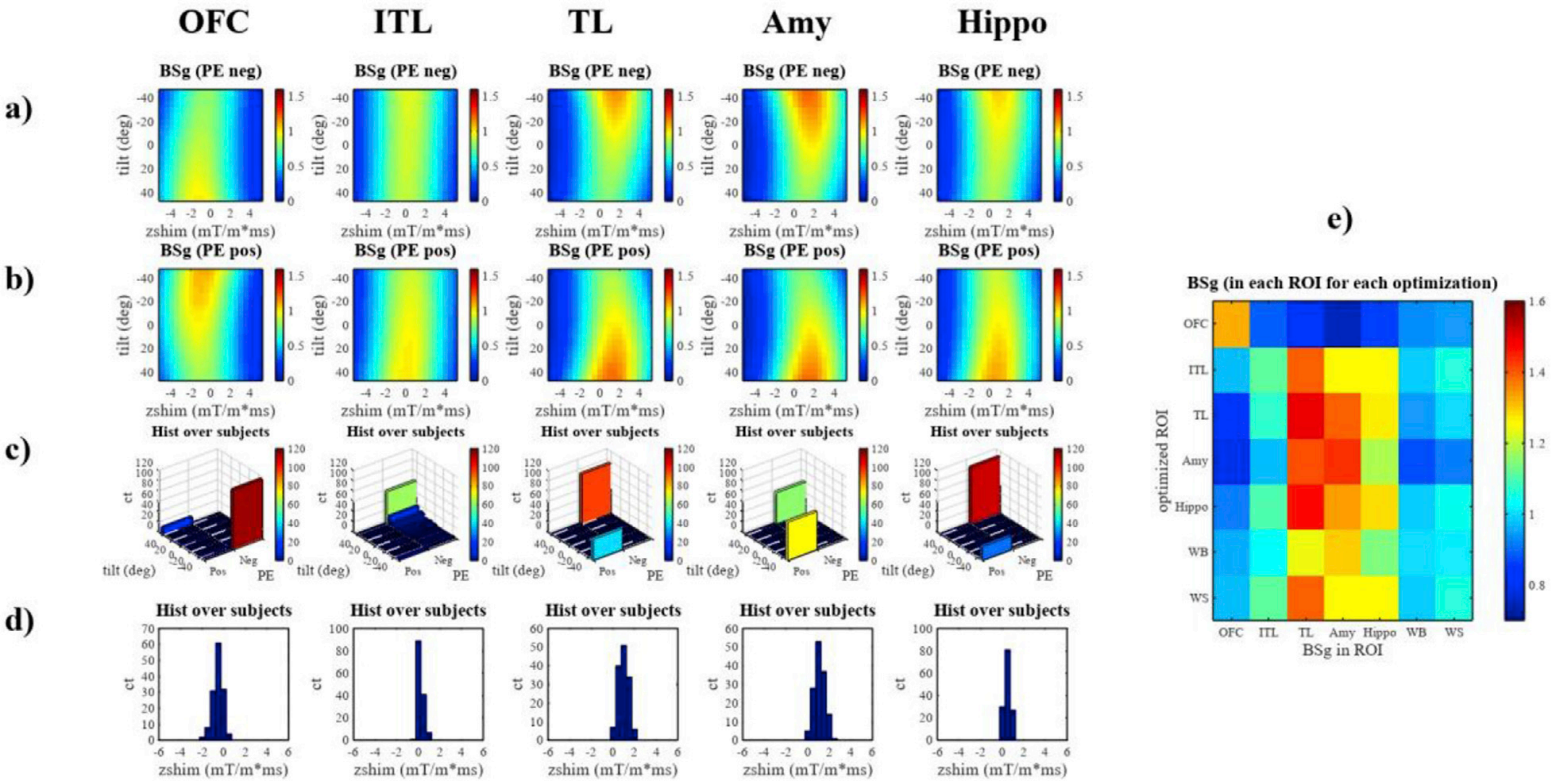
Optimization of the transverse standard resolution protocol for different ROIs: OFC = mOFC + rACC, ITL = Inferior temporal lobes, TL = Temporal lobes, Amy = Amygdala, and Hippo = Hippocampus + Parahippocampus. For the optimization based on group-average field maps the BS gain depending on slice tilt and z-shim assuming either a negative PE direction (a) or a positive phase encoding direction (b) are shown. To convey how well the optimal parameters derived from group-average fieldmaps translate to single subjects, the histograms in (c) and (d) show for how many subjects a particular value of slice tilt and z-shim would result in the maximal BS gain based on individual field maps. The number of subjects for the optimal slice tilt are displayed for each PE direction separately. (e) shows how the optimization for one ROI affects the BS in the other ROIs. As additional ROIs the Whole Brain (WB) and Well Shimmed areas (WS) are shown.

**Fig. 7:**
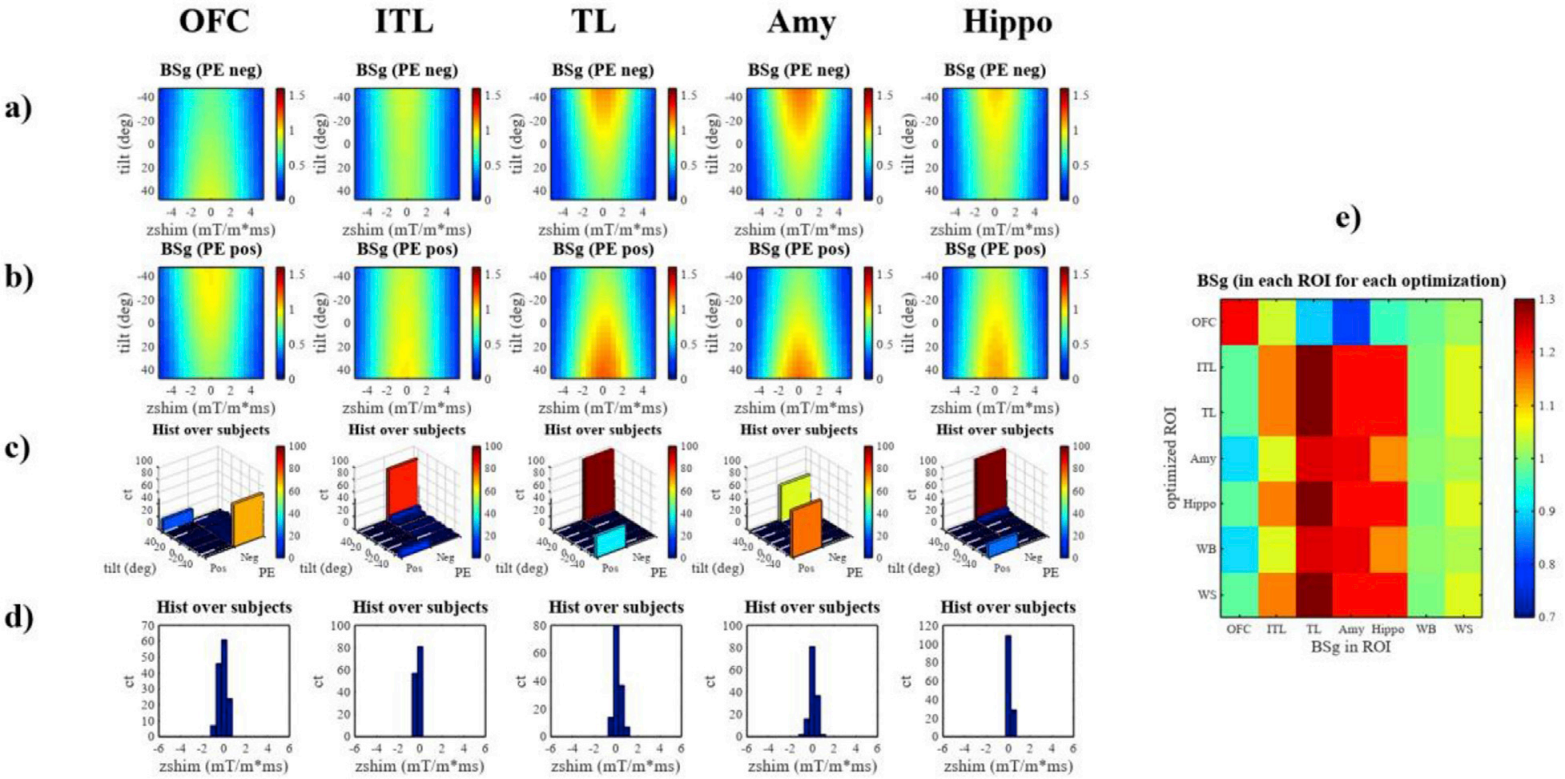
Optimization of the sagittal standard resolution protocol for different ROIs: OFC = mOFC + rACC, ITL = Inferior temporal lobes, TL = Temporal lobes, Amy = Amygdala, and Hippo = Hippocampus + Parahippocampus. For the optimization based on group-average field maps the BS gain depending on slice tilt and z-shim assuming either a negative PE direction (a) or a positive phase encoding direction (b) are shown. To convey how well the optimal parameters derived from group-average fieldmaps translate to single subjects, the histograms in (c) and (d) show for how many subjects a particular value of slice tilt and z-shim would result in the maximal BS gain based on individual field maps. The number of subjects for the optimal slice tilt are displayed for each PE direction separately. (e) shows how the optimization for one ROI affects the BS in the other ROIs. As additional ROIs the Whole Brain (WB) and Well Shimmed areas (WS) are shown.

**Fig. 8:**
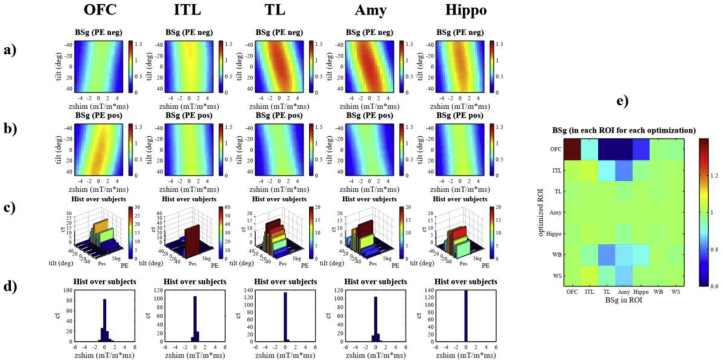
Optimization of the coronal standard resolution protocol for different ROIs: OFC = mOFC + rACC, ITL = Inferior temporal lobes, TL = Temporal lobes, Amy = Amygdala, and Hippo = Hippocampus + Parahippocampus. For the optimization based on group-average field maps the BS gain depending on slice tilt and z-shim assuming either a negative PE direction (a) or a positive phase encoding direction (b) are shown. To convey how well the optimal parameters derived from group-average fieldmaps to single subjects, the histograms in (c) and (d) show for how many subjects a particular value of slice tilt and z-shim would result in the maximal BS gain based on individual field maps. The number of subjects for the optimal slice tilt are displayed for each PE direction separately. (e) shows how the optimization for one ROI affects the BS in the other ROIs. As additional ROIs the Whole Brain (WB) and Well Shimmed areas (WS) are shown.

Fig. 6, Fig. 7, Fig. 8 provide more details for the ROI optimization in addition to Table 1 by showing the BS gain distribution depending on the optimized parameters. Two types of optimization were performed: one based on the group-average fieldmap and the other one based on individual subject fieldmaps. The BS gain for each ROI (columns from left to right) of Table 1 is shown for all combinations of slice tilt and z-shim in the colour maps assuming either a negative PE direction (a) or a positive phase encoding direction (b). Similarly, for each ROI the frequency of the optimal parameters slice tilt and z-shim is shown in the histograms (c) and (d) respectively in case of the optimization based on the individual field maps. In case of (c) the number of simulations resulting in a given optimized slice tilt is displayed for each PE direction separately. This representation was chosen since an opposite PE direction also results in optimized slice tilts with opposite sign. In (d) the number of simulations resulting in a given optimized z-shim value is displayed for both PE directions combined. In addition, the impact of an optimization for each single ROI on the BS of all other ROIs is shown in the coloured checkerboard in (e). Fig. 6, Fig. 7, Fig. 8 show the results for the optimization of the transverse, sagittal, and coronal standard resolution protocol respectively.

In case of the optimization of the transverse protocol a clear bimodal contribution of BS depending on the slice tilt can be observed for both PE directions with a strong preference for the maximum slice tilt (Fig. 6a–b): a change of the PE direction results in an opposite sign of the optimal slice tilt. The subject by subject analysis in Fig. 6c shows that especially for the Temporal Lobes and the Amygdala there was no clear preference for the negative PE with −45° slice tilt or the positive PE with 45° slice tilt parameter set. The contribution of optimal z-shim values is rather sharp with almost all subjects within an interval of ±0.5 mT/m*ms around the maximum (Fig. 6d). The effect of optimizing a protocol for one ROI on other ROIs (Fig. 6e) shows that optimizing the OFC results in a reduction of the BS in all other ROIs, especially in the Amygdala (BS loss of 26%), while the optimization of all other ROIs also increases the BS of all ROIs except for the OFC. The optimization of the Amygdala in turn yields a rather strong BS loss of 21% in the OFC. Additionally, the strong z-shim gradient suggested for the Amygdala, results in a reduced BS of the well shimmed areas and therefore for the whole brain (BS losses 8% and 12% respectively).

Due to the same PE direction for both transverse and sagittal acquisition the results for the optimization of the sagittal protocol shown in Fig. 7 in terms of slice tilt and PE direction are similar to Fig. 6. However, due to the antisymmetric distribution of susceptibility-induced field inhomogeneities in the left-right direction, the optimal z-shim values are centred around zero.

In case of the optimization of the coronal protocol (Fig. 8), a clear preference for one PE direction (negative for OFC and positive for all other ROIs) with a rather broad distribution of optimal slice tilts can be seen in contrast to transverse orientation. While the optimal z-shim values were centred sharply about zero in the subject by subject analysis, it became a function of slice tilt in the simulated parameter space, especially in the Temporal Lobes and Amygdala.

The results of the optimization in different ROIs for different resolutions (3 mm and 2 mm isotropic) and using GRAPPA, factor 2 with 2 mm isotropic resolution for oblique transverse acquisition are listed in Table 2 and show comparable results for the optimized parameters z-shim, slice angulation and PE gradient polarity. The respective BS gains are higher for the 2 mm isotropic compared to the 3 mm isotropic resolution probably due to the fact that with a higher in-plane resolution, and thus a longer acquisition time, the susceptibility gradients in the PE direction result in greater BS losses potentially being compensated by the use of the optimal parameters. The BS gain using GRAPPA, factor 2 is lower compared to using no GRAPPA. This is probably due to less signal losses from susceptibility gradients in PE direction due to the shorter acquisition window.

**Table 2:** Optimal parameters for transverse orientation for different resolutions and when using parallel imaging. (I) in-plane resolution of 3 × 3 mm^2^, matrix size of 64 × 64 and slice thickness of 3 mm, (II) in-plane resolution of 2 × 2 mm^2^, matrix size of 96 × 96 and slice thickness of 2 mm and (III) parameters as in (II), using parallel imaging with GRAPPA, factor 2. As a measure of generalizability, the standard deviation for the BS-gain across subjects is listed.

Region of interest	Z-shim [mT/m*s]			Tilt [deg]			PE polarity			BS-gain [%]		
Acquisition Protocol	I	II	III	I	II	III	I	II	III	I	II	III
mOFC + rACC	−0.5	−1.0	−0.5	−45	−45	−45	Neg	Neg	Neg	37 ± 17	42 ± 20	37 ± 18
Inferior temporal lobes	0.0	0.5	0.5	45	45	45	Neg	Neg	Neg	12 ± 6	15 ± 9	19 ± 9
Temporal poles	1.0	1.5	1.0	45	45	45	Neg	Neg	Neg	44 ± 17	55 ± 21	51 ± 19
Amygdala	1.5	1.5	1.5	−45	−45	−45	Pos	Pos	Pos	44 ± 23	50 ± 25	47 ± 21
Hippocampus + Parahippocampus	0.5	0.5	0.5	45	45	45	Neg	Neg	Neg	30 ± 9	42 ± 13	39 ± 12

### 3.3 Validation by comparison to published approaches

A comparison of optimal parameters determined for different ROIs resulting from the simulations and by doing the optimization with multiple EPI acquisitions according to Weiskopf et al. (2006) are shown in Table 3. The parameter optimization resulted in values of the z-shim, slice angulation and PE gradient polarity being in good agreement for the mOFC and rACC, inferior temporal lobes, temporal poles and the amygdala. However, for the hippocampus the tilt pointed in the opposite direction as did the PE gradient polarity. This can be explained with similar BS gains for parameter sets with opposing slice tilts and PE gradient polarity respectively for the hippocampus region.

**Table 3:** Comparison of simulation based BS optimization with literature. (a) Results of the simulation-based optimization in this study and (b) previous optimization using data from multiple EPI acquisitions (Weiskopf et al., 2006). As a measure of generalizability, the standard deviation for the BS-gain across subjects is listed.

Region of interest	Z-shim [mT/m*s]		Tilt [deg]		PE polarity		BS-gain [%]	
Study	a)	b)	a)	b)	a)	b)	a)	b)
mOFC + rACC	−1	−1.4	−45	−45	Neg	Neg	20 ± 10	19
Inferior temporal lobes	0	−0.4	30	30	Neg	Neg	11 ± 4	4
Temporal poles	1	0.6	45	30	Neg	Neg	25 ± 10	18
Amygdala	1	0.6	−45	−45	Pos	Pos	23 ± 12	13
Hippocampus + Parahippocampus	1	0.6	45	−45	Neg	Pos	18 ± 6	11

### 3.4 Validation by comparison to in vivo data

**Fig. 9:**
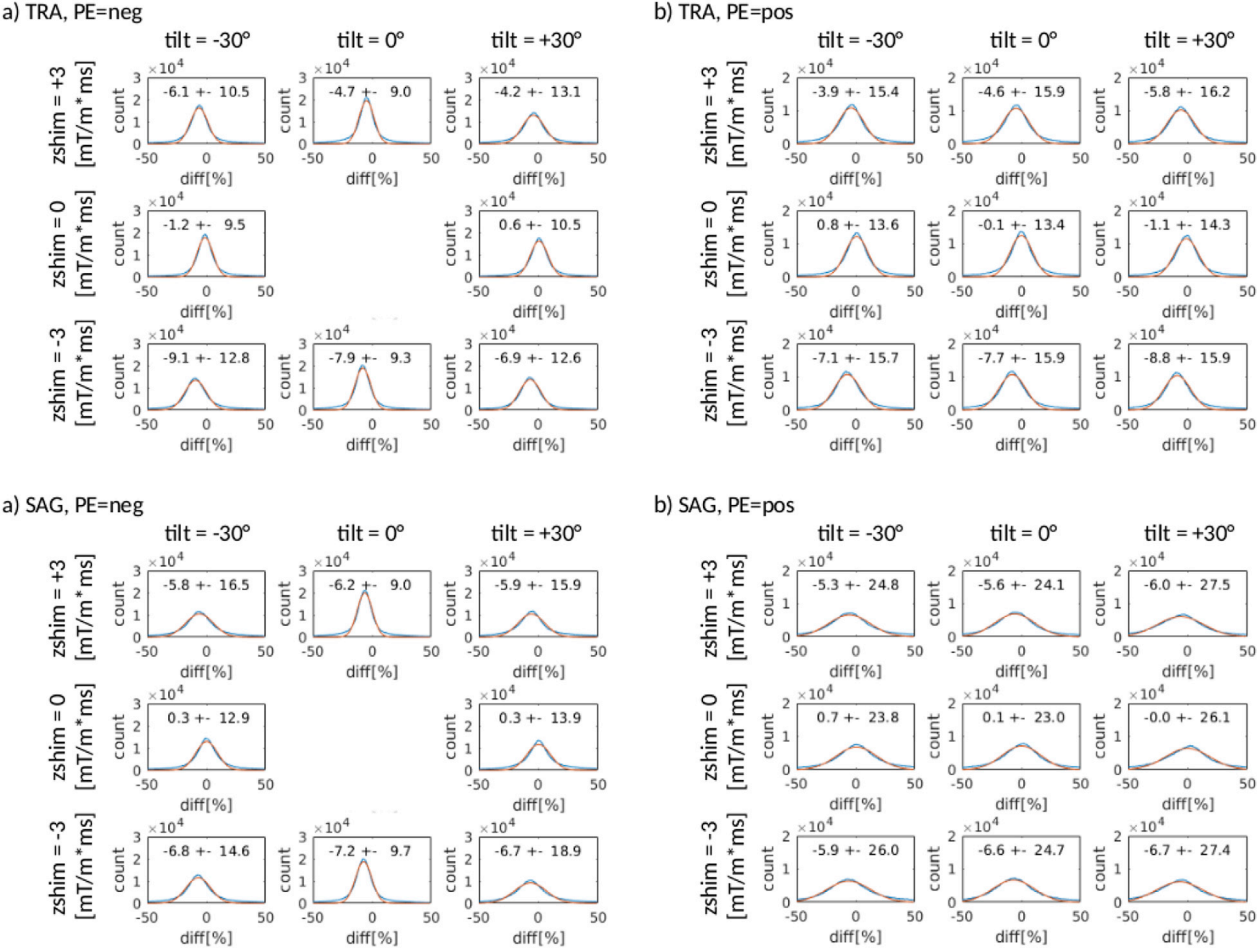
Histograms of the percent deviations between simulated and experimentally measured BS gains pooled across the brain mask (for each of the 36 protocols and six subjects). The optimization results for the transverse protocol are shown for the negative and positive PE direction in a) and b), respectively. The optimization results for the sagittal protocol with negative and positive PE direction are shown in c) and d). The protocol with negative PE and no tilt and z-shim is not shown, since it was the reference protocol. The blue histogram represents the experimental data, while the red curve is a Gaussian fit with the respective estimated mean and standard deviation.

The comparison between simulated and experimental BS gains showed good agreement for each of the 36 protocols investigated (Fig. 9). Deviations were typically around 5% and did not exceed 10%, which is within the standard deviation across the brain mask, which ranges from 10 to 20%. Histograms of the deviations pooled over the brain mask and all subjects showed a Gaussian distribution, suggesting that deviations were largely driven by noise rather than systematic bias due to poor BS model performance.

## 4 Discussion

In this study we presented a flexible and automated BS optimization method based on numerical simulations of the BS loss using physical models that account for the effects of susceptibility-induced gradients in the through-plane (Deichmann et al., 2003), phase-encoding (Deichmann et al., 2003; De Panfilis and Schwarzbauer, 2005) and readout direction (Weiskopf et al., 2007) and informed by a database of magnetic (B0) field maps over a large population of 138 volunteers. The simulations produced results that are in good agreement with earlier experimental optimization outcomes (Weiskopf et al., 2006) and the predicted BS increases are in line with the experimental measures of BS in six volunteers.

Previous optimizations have been based on atlases derived from multiple EPI acquisitions making them resource and time consuming. Consequently, they were performed only for a limited number of parameters over a restricted range and by acquiring data from a small number of volunteers only, e.g. optimizing the z-shim, the gradient polarity and the slice tilt for oblique transverse slices and a resolution of 3 mm on five volunteers only (Weiskopf et al., 2006). Results from these experimental studies suggested, for example, the use of a strong positive slice angulation in temporal regions but a strong negative angulation in orbitofrontal regions, i.e., tilting the slice downwards or upwards at the front (see Fig. 1 for definition of slice angles). These results are confirmed by our simulations when considering the case of oblique transverse slices as used in the original experimental investigation. Extending the optimization to coronal slices, shows that for some regions like the temporal poles coronal slices would be the better choice with respect to BS.

The advantage of the proposed method is that the optimization of parameters is done by simulations thus avoiding time and resource consuming measurements. This also allows for the optimization to be performed over a larger parameter space including varying resolution, echo time or slice orientation. The parameters that are optimized and the range of optimization can easily be adapted without the need for additional measurements. In addition, various boundary conditions can be readily implemented in the optimization (e.g. preferred slice tilts for specific anatomical coverage). Although here the optimization yielded a single set of parameters (slice angulation, z-shim and PE direction) to be applied for all slices, optimized parameters could in principle be varied from slice to slice, e.g. allowing for slice dependent TE (Domsch et al., 2013) and z-shim (Rick et al., 2010; Bonnici et al., 2012; Finsterbusch et al., 2012). Our results indicate that such an approach would be of benefit in regions such as the temporal lobes where both positive and negative susceptibility gradients are in close spatial proximity requiring opposing optimal parameter settings to maximise BS. Especially in case of coronal or sagittal acquisitions we found a left-right antisymmetric distribution of the field inhomogeneities and correspondingly opposing values for the optimal shim gradients (Fig. 2, Fig. 3). This results in near zero z-shim values for the ROI optimisation (Table 1) in case of one optimal parameter set valid for all slices and ROIs with voxels being distributed symmetrically on the left and right hemisphere of the brain. In this case slice-specific z-shims could yield better results if available.

Although coronal slices may yield a higher BS in temporal areas, the slice orientation affects the brain coverage that can be achieved with a specific TR and slice thickness as well. Coronal slices compared to transverse slices have the disadvantage of a lower brain coverage at the same TR and require a longer TR if whole brain coverage is needed, i.e. an increase in scan time of about 30–35% (Mennes et al., 2014). Similarly, the slice thickness affects the number of slices necessary to cover a specific region and hence the needed TR and hence limit the parameter range or prescribe specific parameters if necessary. Therefore, a compromise has to be found and the user must decide on the necessary coverage and maximum TR. For example, the study of the amygdala typically requires high spatial and temporal resolution as well as extended coverage in order to account for its small dimensions while concurrently allowing its activation to be interpreted within a complex network of brain regions (Stöcker et al., 2006).

The use of the slice tilt can also be limited by the gradient performance, since gradients in different directions contribute differently to peripheral nerve stimulations. Also when applying gradients in different directions with respect to the slices at the same time, the real gradients played out on the scanner can be higher due to vectorial summation and gradient amplitudes as well as slew rates may exceed hardware specifications, if already the reference protocol was near the limit. This means that either a reduced gradient performance with a reduced temporal resolution has to be accepted, to ensure the setup of the optimized protocol at the scanner, or the range of possible parameter settings has to be limited for the simulation and optimization. Also, in case of extreme magnetic field variations as in the vicinity of metallic implants, the proposed approach is likely to underperform or fail due to induction of eddy currents, requirement of excessive z-shim gradient moments or issues with the RF excitation.

Additionally, the simulation only optimizes the BS. Temporal SNR or potential artefacts have to be additionally taken into account when defining the parameter space for optimization. For example, parallel imaging on the one hand increases the acquisition speed allowing for a lower TR, important e.g. for high resolution imaging of the whole brain. A short TR can also be used to reduce physiological noise by removal of high frequency noise originating from respiration and cardiac pulsation (Todd et al., 2017). On the other hand it reduces the overall SNR and poses a potential source of artefacts arising from g-factor penalties and issues with reference scans in combination with volunteer movement (Feinberg and Setsompop, 2013). New imaging techniques like simultaneous multislice imaging offer the possibility of accelerating the imaging without direct loss of SNR. However, still SNR loss occurs due to unfavourable g-factors and slice leakage artefacts occur in combination with in-plane acceleration making the overall statistical benefit dependant on the used hardware and region of interest and requiring a careful consideration for choosing the best acceleration factor (Todd et al., 2016).

The optimization of an EPI protocol for a specific region often comes with the reduction of the BS in other regions. In this study the BS loss in areas not affected by susceptibility related gradients, i.e., well shimmed areas, was limited to 15%. However, the BS loss may be significantly higher in areas requiring different optimal parameters due to susceptibility induced field gradients pointing in opposite direction compared to the optimized areas (Weiskopf et al., 2006). While the TE or z-shim could be optimized slice-wise, provided that appropriate sequences are available, the slice tilt can be optimized only for a single or combined ROI.

The field maps used for the numerical simulations of the BS in this study were acquired at 3T on a Siemens Tim TRIO scanner. Strictly speaking the optimization results are valid only at this field strength and for this scanner type. However, we would expect that this population-based field map gives a good estimate of the susceptibility field distortions introduced by such a scanned object (the volunteer's head and body) in a particular position in a largely-uniform magnetic field. Since the head position and orientation in different MRI scanners are similar (Weiskopf et al., 2006), these optimized parameters can be expected to also hold across different systems to good effect. In addition, the magnitude of these field inhomogeneities would likely scale linearly with the main static magnetic field strength, meaning that even higher BS gains can be expected at higher field strengths. Nonetheless, interaction with the applied shim gradients counteracting the field inhomogeneities mean that this simple relationship is limited making the results dependent on the used hardware, i.e. the type and performance of the shim coils.

The large sample of field maps used for optimization this framework promises to provide an improved optimization for group studies, since the typical distribution of field inhomogeneities in the population is better captured compared to previous experimental optimizations based on few volunteers only. In principle, also a subgroup of field maps (e.g. a specific age range or sex) can be used for optimization. However, for studies on atypical populations, e.g. patients with atypical skulls or brains, the field map database and the optimizations based on it may not be optimal. The flexible framework of the proposed method would allow replacing the correct field map database with a patient group specific database or even allow for using individual field maps. This is also valid in case a new field map data base is needed for a different scanner type as discussed above. A description of how to create a new database is given in the methods section of the manuscript (“*Acquisition of a large magnetic field map database*”).

In this study field maps with a somewhat lower resolution of 3 mm were used. This might be suboptimal for optimizing small structures in a high resolution EPI protocol for a single subject. However, this study aimed to optimize EPI protocols for group studies primarily. Therefore, we can assume that due to inter-subject variability very small structures will be blurred out and do not matter in a group optimization. The optimization for a group and scanning the group with a single fixed parameter set simplifies the experimental workflow significantly and requires only small changes to standard EPI sequences, which facilitates larger population and routine neuroimaging studies.

The optimization of BS is only based on the simplified assumption of constant thermal noise. We neither performed a fMRI experiment nor acquired a time series for determining the temporal SNR, which is central for precise measures for sensitivity. Thus, in the vicinity of contrast edges and air–tissue interfaces with strong susceptibility gradients or near large blood vessels, the real BS may turn out to be lower than expected from the simulations, since head motion or respiration and cardiac movement cause prominent physiological noise contributions, especially at high field strength in combination with large voxel sizes (Krüger and Glover, 2001, Krüger et al., 2001, Triantafyllou et al., 2005; Van de Moortele et al., 2008; Hutton et al., 2011). It was however also shown (Triantafyllou et al., 2005) that for the simulations performed here for 3T and 3 × 3 × 3 mm^3^ resolution that thermal noise dominates over physiological noise with a ratio of about 0.89 and 0.70 for 2 × 2 × 3 mm^3^ suggesting that the simplified SNR model for the simulations is still a relatively good approximation in this regime (Balteau et al., 2010). The simplified noise model also prohibits the direct comparison of BS across different spatial resolution, since both thermal SNR and temporal SNR change in this case. However, these effects are not expected to significantly impact comparisons of different parameter sets (slice tilt, z-shim, PE) at the same TE and resolution. Related to this, we note that we did not perform fMRI experiments to validate the predicted BS gain in the different ROIs, since multiple group experiments with different tasks would have been required and going beyond the scope of this study.

## 5 Conclusion

The presented method allows for automated optimization of arbitrary 2D-EPI protocols based on a population magnetic field map database avoiding expensive measurements that consume time and resources. The basic protocol can easily be changed allowing for optimization over a larger parameter space compared to previous experimental based optimization methods. The large dataset of field maps also promises to provide improved optimization for group studies, since the typical distribution of field inhomogeneities in the population is well captured. The results of the optimization by simulations are in good agreement with earlier experimental optimization outcomes (Weiskopf et al., 2006) and the expected BS increases are in line with experimental BS measurements.
